# Secure Indoor Water Level Monitoring with Temporal Super-Resolution and Enhanced Yolov5

**DOI:** 10.3390/s25092835

**Published:** 2025-04-30

**Authors:** Sui Guo, Jiazhi Huang, Yuming Yan, Peng Zhang, Benhong Wang, Houming Shen, Zhe Yuan

**Affiliations:** 1 China Yangtze Power Co., Ltd., Yichang 443000, China; guo_sui@ctg.com.cn (S.G.); huang_jiazhi@ctg.com.cn (J.H.); yan_yuming@ctg.com.cn (Y.Y.); zhang_peng5@ctg.com.cn (P.Z.); wang_benhong@ctg.com.cn (B.W.); 2 Wuhan NARI Limited Liability Company, State Grid Electric Power Research Institute Co., Ltd., Wuhan 430070, China; yuanzhe@sgepri.sgcc.com.cn

**Keywords:** water level monitoring, image super-resolution, hydropower plants, intelligent security applications

## Abstract

Ensuring secure and efficient water level monitoring is critical for the intelligent management of hydropower plants, especially in challenging indoor environments. Existing methods, which are tailored for open areas with optimal conditions (adequate lighting, absence of debris interference, etc.), frequently falter in scenarios characterized by poor lighting, water vapor, and confined spaces. To address this challenge, this study introduces a robust indoor water level monitoring framework specifically for hydropower plants. This framework integrates a temporal super-resolution technique with an improved Yolov5 model. Specifically, to enhance the quality of indoor monitoring images, we propose a temporal super-resolution enhancement module. This module processes low-resolution water-level images to generate high-resolution outputs, thereby enabling reliable detection even in suboptimal conditions. Furthermore, unlike existing complex model-based approaches, our enhanced, lightweight Yolov5 model, featuring a small-scale feature mapping branch, ensures real-time monitoring and accurate detection across a variety of conditions, including daytime, nighttime, misty conditions, and wet surfaces. Experimental evaluations demonstrate the framework’s high accuracy, reliability, and operational efficiency, with recognition speeds reaching O(n). This approach is suitable for deployment in emerging intelligent systems, such as HT-for-Web analysis software 0.2.3 and warning platforms, providing vital support for hydropower plant security and emergency management.

## 1. Introduction

Secure and efficient water level monitoring is essential for the intelligent management of hydropower plants and plays a key role in improving the reliability and safety of modern water conservation systems [1]. The dispersed and remote nature of hydropower plants often leads to challenges such as information silos and delayed responses in their management [2,3], making real-time monitoring and intelligent control mechanisms critical. Strengthening effective monitoring at all levels, obtaining timely field information to respond quickly to emergencies, optimizing hydropower generation, and integrating safe production with flood control and irrigation needs remain top priorities for government agencies [4,5]. Real-time monitoring of water levels in catchment wells is particularly challenging due to confined indoor spaces, inadequate lighting, and high humidity, especially during the rainy season [2]. These unique environmental conditions exacerbate the difficulty of implementing traditional monitoring methods and underscore the need for innovative, intelligent solutions. Despite advances in technology, real-time monitoring in such complex scenarios (poor lighting, water vapor, and confined spaces, etc.) has yet to be fully solved [6,7].

Traditional methods for real-time monitoring of water levels in the intake wells of hydropower plants are mainly divided into two categories: (1) contact-based (sensors) methods [8,9] and (2) non-contact methods [3,10,11]. Specifically, the contact methods are mainly used by the physical sensors (such as float and pressure group type, etc.) to monitor the water level through the transmission of signals. Despite its simplicity, this type of method is easily affected by clutter in the water, which tends to cause monitoring errors and is easily damaged, resulting in high maintenance costs. Non-contact methods using sensors (e.g., laser, radar, ultrasound, etc.) are not affected by debris in the water, but their construction is complex, the hardware equipment is expensive, and they are difficult to apply on a large scale in the actual hydropower plant scenarios [12]. Recently, with the development of artificial intelligence (AI) and image processing technologies, many scholars have applied image processing with deep learning technologies to water level monitoring in various methods at home and abroad [13,14]. However, their methods are mainly aimed at monitoring water levels under ideal lighting and unobstructed conditions using complex models with extensive parameter tuning. Although promising progress has been made, the performance of the system in the dark and humid environment of hydroelectric power plants has deteriorated considerably [15].

To address the above problem, in this study, we propose a water level monitoring framework with a temporal super-resolution technique and an improved Yolov5 [16] model in hydropower plants for safe applications. Specifically, unlike the existing degradation-based methods for image super-resolution, the former proposed a temporal super-resolution enhancement (scaling) technique, which mainly used resolution temporal scaling factors to process low-resolution water level images to achieve high-resolution outputs. Meanwhile, the latter constructs a small-scale feature mapping branch model, combines with the output of the previous stage, fully connects with the upsampling output of large-scale feature mapping in the original structure, and then jointly performs convolution operations with large-scale and mesoscale features to obtain the final small-scale feature mapping output to ensure the integrity of water level monitoring.

Our method possesses the capability to recognize specific indoor scenes at a velocity of 4500 instances per second, rendering it ideal for deployment in nascent intelligent systems. These include HT-for-Web (HTWEB, https://chinadaily.com.cn/, accessed on 19 December 2024) analysis software 0.2.3 and warning platforms for urgent water-level monitoring in hydropower plants. Furthermore, the intelligent water level monitoring system necessitates the integration of support systems, such as alarm and emergency response mechanisms, along with synchronization with existing monitoring systems. This integration is pivotal in enhancing the safety warnings for water levels in existing hydropower plants and augmenting the efficiency of emergency responses. By incorporating efficient and swift detection algorithms tailored for hydropower plant scenarios into novel intelligent systems, real-time water level changes in these plants can be detected with precision. The method proposed in this study, leveraging Yolov5 as its backbone network, is particularly suited for this application due to its proficiency in detecting water level targets and its relatively low computational resource requirements, thereby facilitating the efficient and rapid completion of detection tasks.

Extensive experiments have been conducted to demonstrate the performance of our proposed method. Meanwhile, the experiments also evaluate the contribution of each submodel of the proposed method to such performance gains through ablation studies and visualization results.

In short, the main contributions of this work can be summarized as follows:

1. We propose a real-time water level monitoring framework, which includes a temporal super-resolution enhancement model and an improved Yolov5 structure.
2. The introduced temporal super-resolution enhancement module adeptly manages varying degrees of super-resolution images, achieving high-definition outputs through a strategy involving temporal scaling factors for resolution. The enhanced Yolov5 architecture is designed with a small-scale feature mapping branch, which subsequently collaborates with large-scale and mesoscale features through convolution operations to produce the ultimate small-scale feature mapping output, thereby ensuring the comprehensiveness of water level monitoring.
3. Extensive experiments are conducted on the self-made datasets, which were collected on-site for water level monitoring. This also demonstrates the practical application significance of our method.

## 2. Related Work

As mentioned above, existing methods based on water level monitoring are mainly divided into contact and non-contact methods. The former mainly uses physical sensors to perform the task, while the non-contact method mainly includes the use of radar, ultrasonic, and image recognition modes. In addition, we also reviewed the existing image super-resolution methods. Representative work is described as follows.

### 2.1. Contact-Based (Sensors) Methods

The contact-based method is mainly based on the Internet of Things (IoT) or physical sensors to complete water level monitoring. Rosolem et al. [17] proposed an optical sensor of water level equipped with a standard single-mode fiber, which measures the water levels up to 10 m or more by choosing the appropriate membrane. Wang et al. [18] derived water level based on the Global Ecosystem Dynamics Investigation (GEDI, International Space Station-based) data with a mobile sensor to remove systematic errors. Miller et al. [6] studied from the perspective of Internet of Things (IoT) technology. It has penetrated various areas of our lives, especially in the application of monitoring natural water levels, which is often associated with complex sensor technology to maximize understanding of changes in water levels in nature. Ranieri et al. [8] monitored the water level by controlling the angle of incidence, distance, and turbidity of the water level through sensors. The model, combined with inertial sensor data, improved the accuracy of water level prediction compared to a single sensor. Salam et al. [10] proposed an intelligent management system for autonomous irrigation of rice fields based on sensor control. By monitoring and controlling water levels in real time, sustainable use of water resources and the development of new technologies can be achieved. In addition, other water level monitoring methods based on hardware sensors have also been widely used [9,19].

### 2.2. Non-Contact-Based Methods

The non-contact method uses mainly radar, ultrasound, and image processing technologies to accomplish this task. Mohammadimanesh et al. [15] proposed a radar-based interactometric synthetic aperture method for wetland hydrological monitoring and systematically reviewed the advantages and disadvantages of current non-contact water level monitoring methods. Xu et al. [20] predicted seasonal water level changes in specific lakes based on Sentinel-3 radar data. It has been confirmed that changes in water level values are influenced by factors such as internal terrain characteristics, lake shape, and area. Zhang et al. [11] presented a method based on ultrasonic measurement of liquid level in wooden barrels. This method uses an ultrasonic probe that slides on the surface of the container and combines the different ultrasonic acoustic properties of liquid and air to measure the height value of the liquid in the bucket. In addition, methods based on image processing and deep learning have developed rapidly in recent years. Among the representative works, Cheng Shuhong et al. [4] proposed a method of water level line identification using U-net image segmentation technology. Through the U-net water level automatic segmentation technique, the water level line can be accurately labeled, and the influence brought about by the image background in the process of water level measurement is solved. In addition, Zhuo et al. [5] used an SSD target detection algorithm for the study of video water level detection. Wang et al. [21] proposed a real-time water level recognition algorithm consisting of Yolov3 object detection and a Resnet network [22] scale recognition model; the method water level recognition progress will continue to improve, and has the application space to expand to the detection of the section site environment recognition. The method can accurately locate the water scale target and extract the corresponding water level values. These studies provide new ideas and methods for the development of water level detection technology.

### 2.3. Image Super-Resolution Methods

Image super-resolution (SR) refers to the process of restoring high-resolution (HR) images from low-resolution (LR) images [23,24], and it has a wide range of applications in medical imaging [25], virtual reality [26,27], and other fields. Li et al. [23] proposed a RealFuVSR model that simulates real-world degradation to mitigate artifacts caused by video super-resolution, which helped eliminate hidden state artifacts. Ye et al. [27] proposed a neural accumulator based on heterogeneous super-resolution to recursively aggregate and amortize low-resolution visual information for real-time rendering of inherent dynamic and geometric information in the content frame by frame. Zhu et al. [24] proposed a video super-resolution task learning based on a frame-by-frame feedback fusion network, which achieved high-definition video reproduction by rearranging video frames based on their distance from the reference frame in time and space. Of course, there are also other applications of super-resolution task learning in water level monitoring, which will not be listed here.

Although existing methods have, indeed, made some progress in water level monitoring, they are mainly targeted at outdoor scenarios with sufficient light: relatively ideal environments [28]. Differently from them, this work proposes a water level monitoring model based on time-enhanced image super-resolution and improved Yolov5 (You Only Look Once version 5) to realize water level monitoring in the internal scenes of hydropower.

## 3. The Proposed Method

### 3.1. Overview

In this section, we mainly introduce the method proposed in this paper, which is based on temporal super-resolution enhancement and an improved Yolov5 water level monitoring method. Firstly, we mainly introduce the super-resolution model based on temporal enhancement. Secondly, for the output after super-resolution, we use the improved Yolov5 for water level recognition. The specific details are as follows.

### 3.2. Temporal Super-Resolution Enhancement Model

Image super-resolution (SR) [13,27] refers to the process of recovering high-resolution (HR) images from low-resolution (LR) images, and is an important image processing technique in the field of computer vision and image processing, which has a wide range of real-world applications, such as medical imaging [25] and surveillance security [23,24]. Typically, digital images are composed of pixels, and the pixels of a high-resolution image are generated from the pixels of a low-resolution image by super-resolution mapping [24,29]. Therefore, the purpose of image super-resolution is to find the perceptual error between the high-resolution image and the low-resolution image by generating (reconstructing) the high-resolution image through a generator [30,31]. Existing image-based super-resolution methods, as cited in [30], primarily rely on the degenerate fuzzy model. The specific approach involves mapping the high-resolution (HR) image space to the low-resolution (LR) image space. While these methods have demonstrated impressive performance, their primary focus is on conducting super-divisional loss perception within the LR image space. However, due to the inherent pixel deficiency in actual LR images, the direct degenerate fuzzy approach to error mapping inevitably leads to further pixel loss, thereby impacting the overall performance. In response to the shortcomings of existing super-resolution methods, this study proposes an image super-resolution technique based on temporal enhancement (or scaling). This technique primarily employs a resolution temporal scaling factor to control the varying degrees of super-resolution in the images. In addition, inside actual hydropower stations, the lighting conditions are characterized by fluctuations, exhibiting a dynamically changing state. Given that temporal super-resolution preprocessing features gradual image enhancement, it is ideally suited for such scenarios. The specific details are described below.

Given an LR→HR image pair denoted as (.), where HR and LR are the high- and low-resolution states of the ith image, respectively, the resolution output control factor is s, which is used to control different degrees of image resolution. Referring to [32,33,34], we define a forward temporal diffusion model *D*, denoted as Equations (1) and (2).

(1) $$D\left(y_{1:T}|y_{0}\right)=\sum_{t=1}^{T}D(y_{t}|y_{t-1}),$$

(2) $$D\left(y_{t}|y_{t-1}\right)=\phi \left(yt|{\sqrt{1-\beta }}_{t}y_{t-1},\beta_{t}I\right),$$

where $\beta_{t}\in \left(0,1\right)$ is the Gaussian noise, *T* is the number of timing iterations, *I* is the unit matrix, and ϕ(·) is the mapping model. Given an image $y_{0}$, the value $y_{t}$ at time *t* is given as Equation (3).

(3) $$D\left(y_{t}|y_{0}\right)=\phi \left(y_{t}|\sqrt{\gamma_{t}y_{0}},\left(1-\gamma_{t}\right)I\right),$$

where $\gamma_{t}=\prod_{i==1}^{t}\left(1-\beta_{i}\right)$. In the hypersegmentation inverse process, $p_{\theta }\left(y_{t-1}|y_{t},x\right)$ is set as the learning conditional distribution, θ as the conditional parameters, and the potential features are denoised sequentially during the training process. Similarly, the inference process can be performed in reverse chronological order [35,36], i.e., the reverse chronological mapping from Gaussian noise $y_{T}∼\phi \left(0,I\right)$ to target image $y_{0}$ is represented as Equations (4)–(6).

(4) $$p_{\theta }\left(y_{0:T}|x\right)=p\left(y_{T}\right)\prod_{t=1}^{T}p_{\theta }\left(y_{t-1}|y_{t},x\right),$$

(5) $$p\left(y_{T}\right)=\phi \left(y_{T}|0,I\right),$$

(6) $$p_{\theta }\left(y_{t-1}|y_{t},x\right)=\phi \left(y_{t-1}|u_{\theta }\left(x,y_{t},t\right),\sigma_{t}^{2}I\right),$$

Specifically, the image super-resolution algorithm focuses on inferring a series of denoising steps from a given target image $y_{0}$ to obtain a high-definition image [23], that is, the denoising model $f_{\theta }$, which is equivalent to recovering the target image ${\hat{y}}_{t}$ from a noisy image (low-resolution image) [37]. The specific expression is as Equation (7).

(7) $${\hat{y}}_{t}=\sqrt{\gamma_{t}}y_{0}+\sqrt{1-\gamma_{t}}f,$$

where *f* is the noise prediction model, f∼ϕ(0,I), while to obtain a continuous hypersegmented image output, then the actual noise model $f_{\theta }\left(x,t,s,{\hat{y}}_{t},\gamma_{t}\right)$, the whole hypersegmentation optimization process is as Equation (8).

(8) $$L_{r}=L_{\left(x,y\right)}=||f-f_{\theta }\left(x,t,s,{\hat{y}}_{t},\gamma_{t}\right){\left|\right|}_{1}^{1},$$

where “${\left||\;|\right|}_{1}^{1}$” denotes the vector difference loss calculation, and t∼{1,⋯,T}, s∼u(1,M) is a uniformly distribution, which is drawn from a uniform distribution with a lower bound of 1 and an upper bound of M. (x,y) denotes the pair of low and high resolution.

### 3.3. Improved Yolov5 Structure

Yolov5 (You Only Look Once version 5), as an advanced real-time target detection algorithm, has been widely used in water level image analysis. Yolov5 not only has excellent detection speed and accuracy, but also further improves the detection performance in complex scenes through its unique multi-scale feature fusion mechanism. Usually, the Yolov5 network model mainly consists of input, backbone, neck, and head structures. Among them, the input side mainly adopts Mosaic data enhancement [38] and adaptive anchor frame computation [39], which performs preprocessing operations such as cropping, scaling, flipping, and splicing on the captured image to form a new image. Mosaic’s adaptive anchor frame can automatically adjust the size of the target frame to match the size of different targets. Yolov5’s backbone mainly adopts New CSP-Darknet53 [25] as the backbone network structure, which effectively improves the model computation efficiency and feature acquisition capability. Due to the incorporation of cross-stage partial connections, it can minimize computational redundancy and enhance the efficiency of practical application and deployment, particularly in real-time water level monitoring scenarios. In addition, CSP-Darknet53 introduces a spatial pyramid pooling module that effectively manages multi-scale features, making it a more viable option than other models for our enhanced Yolov5. Specifically, the neck part adopts FPN (Feature Pyramid Network) [40] and PAN (Path Aggregation Network) [41] structures for up- and downsampling strategies to achieve the feature extraction capability. The head part follows the structure of Yolov3 to ensure its performance [42]. In addition, FPN constructs both bottom-up and top-down feature fusion pathways to integrate feature maps of varying scales, generating feature pyramids rich in multi-scale information. This enhances the performance of small object detection, aligning perfectly with the requirements of water level recognition in our task. PAN employs adaptive feature pooling technology, enabling each candidate region to acquire information from all feature layers, thus avoiding reliance on a single calibrated feature layer. This improves the integrity and diversity of the information obtained. While FPN fusion of multi-scale features prevents the output feature dimension from becoming excessively high, PAN’s feature pooling technique retains crucial and significant features while reducing computational complexity. Therefore, the collaborative optimization of these two components effectively achieves the detection of water level lines.

**Figure 1:**
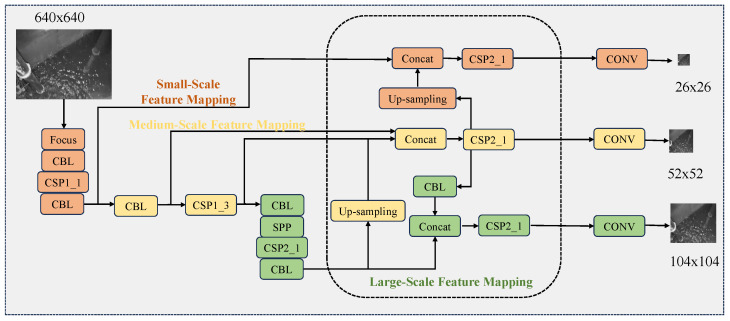
Improved Yolov5 structure.

Meanwhile, due to the unique characteristics of catchment well water levels in hydropower plants—namely, their small granularity, narrowness, and ambiguous boundaries—the conventional Yolov5-based model, primarily utilized for detecting steel-featured targets, inevitably experiences performance degradation when directly applied to water level detection. Furthermore, in the actual indoor monitoring environment of hydropower stations, distinguishing the water level line from the surrounding environment is challenging due to the minimal contrast between them. Traditional features based on conventional scales often struggle to capture the subtle differences between the water level line and its surroundings. Therefore, it is imperative to adopt small-scale feature extraction methods to extract highly discriminative and significant features from the image. Consequently, this study introduces a small-scale feature mapping branching model, which is anchored on the CBL output of the second stage. This model is fully integrated with the upsampled output of the large-scale feature mapping in the original structure, and subsequently undergoes a convolution operation via CSP2_1 to yield the final small-scale feature mapping output. The specific structural framework is illustrated in Figure 1. It is evident that the enhanced Yolov5 model proposed in this study primarily comprises three scales of feature mapping: small-scale, medium-scale, and large-scale, spanning across three dimensions. For the input size of 640×640 images, and through the feature mapping and linking of three different scales to each other, three different resolution output features, i.e., 26×26, 52×52 and 104×104 multidimensional features, and these three features are jointly trained to complete the final water level recognition. We further explain the meanings of the corresponding modules in Figure 1. Among them, “CONV” denotes the convolution operation, “BN” denotes the batch normalization, “Leaky relu” denotes the linear activation function, “Concat” denotes the fully connected operation, “CSP_x” denotes x residual layers to follow, “SPP ” denotes the spatial pyramid pooling operation, and “Res Unit” denotes the residual structure unit. “Slice” denotes the segmentation of the feature map, and “Max pooling” is the maximum pooling operation.

As in the specific model training, we follow the existing loss function based on Yolov5 to complete the model training, in which the Yolov5 model mainly has classification loss and GIoU loss function [43]. In the specific water level detection process in this work, the first step is to detect the water level boundary line, and then carry out the corresponding numerical processing [44], so this work mainly uses the classification loss function to complete the corresponding model training, and its specific process is expressed as Equation (9).

(9) $$L_{cls}=-\frac{1}{N}\sum_{i}^{N}\left[q_{i}\log\left(p_{i}\right)+\left(1-q_{i}\right)\log\left(1-p_{i}\right)\right],$$

where $q_{i}$ is the *i*-th ground truth label and $p_{i}$ is the *i*-th prediction probability. *N* is the size of the samples. In this process, the corresponding water level value is detected by the Yolov5 model, and the actual water level is predicted after focusing on the coordinates.

After the Yolov5 benchmark network extracts the water level image, it will output three scales of feature mappings: large, medium, and small, as shown in Figure 1. Among them, large-scale feature mapping contains the least number of bottom-layer features and the most high-level features; small-scale feature mapping contains the most bottom-layer features and the least high-level features; and mesoscale feature mapping has both. Yolov5 fuses the three scales of feature mapping by using the FPN [40] plus PAN [39,41] feature fusion module, which provides excellent target detection performance. The water level line has the characteristic of being infinitely large in its extension direction and infinitely small in other directions.

### 3.4. Model Optimization

In the process of water level monitoring, whether the target localization is accurate or not directly affects the detected water level results [45]. Therefore, it is necessary to improve the ability of Yolov5 for feature mapping to convey spatial information and detail information [2]. In specific operations, this work is mainly based on the method of image super-resolution [24] and improved Yolov5 model to complete the detection of water level. The whole framework model is optimized in an “end-to-end” manner, and the whole framework is used to complete the whole loss function training. The loss function *L* is shown as Equation (10).

(10) $$L=\left(1-\lambda \right)L_{r}+\lambda L_{cls}.$$

where λ∈(0,1) is a control parameter to prevent the underfitting of the model during the training process in favor of image super-resolution enhancement or target detection, to achieve its control of the overall framework of training the balance, and, at the same time, to ensure that the model of the role of lightweight and efficient.

## 4. Experimental Results and Analysis

### 4.1. Datasets

**Figure 2:**
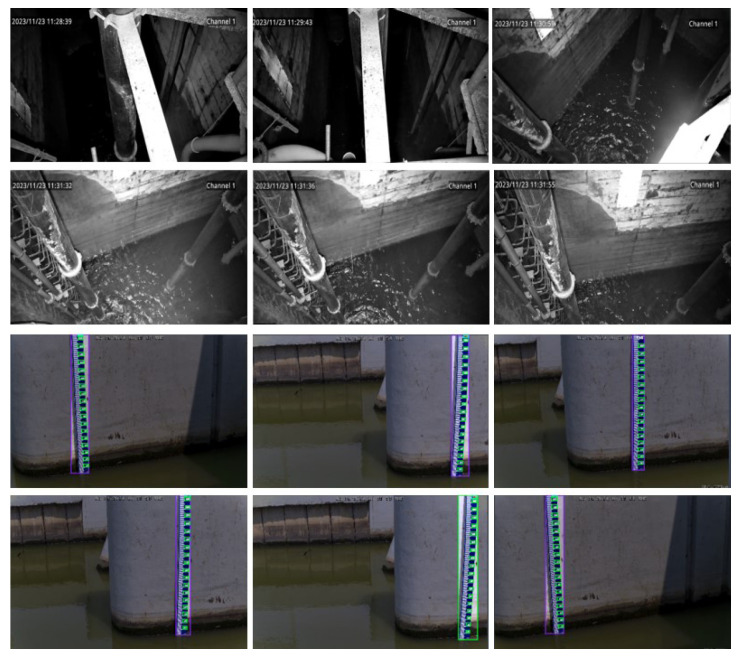
Examples of the dataset. It specifically presents the water level variations in both indoor and outdoor scenarios. The first and second rows depict the scene distribution inside the sump well of a hydropower station, while the third and fourth rows show the water level variation for outdoor scenarios.

A total of 5000 water level images were collected in the field, spanning various time periods. Specifically, 1800 images depict daytime scenes, 2000 represent night scenes, 1000 showcase water mist scenes within wells, and 200 capture wet edge scenarios. Upon observing these images, it becomes apparent that daytime scene images exhibit high clarity. However, the presence of numerous shadows in the images due to lighting conditions often obscures the water level area, posing certain challenges for target detection. Conversely, due to the utilization of infrared acquisition at night, the clarity of night images is not as high. Furthermore, factors such as infrared overexposure significantly impacted the image quality. Subsequently, the water level line was labeled, and the labeled images were proportionally divided into a training set comprising 4000 images and a test set of 1000 images. To mitigate the risk of overfitting, the training set was augmented to include 15,000 images. The collected dataset samples are shown in Figure 2.

### 4.2. Evaluation Metric

For ease of measurement, the marker location must be above the water body and must not be obscured by the water body. The actual water level elevation [1] is then solved as shown using Equation (11).

(11) $$H_{\mathrm{real}}=h^{\prime }-kl=h^{\prime }-\frac{r}{p}l.$$

where $h^{\prime }$ denotes the height of the marker, *p* denotes the pixel length of the marker in the image in pixels, and *l* is the scale of 1:1 ratio. *r* denotes the actual length of the marker, and the unit is meters.

Typically, the performance of target detection algorithms is assessed using metrics such as average precision (AP), mean average precision (mAP), accuracy, and recall. However, the water level line exhibits unique characteristics: it is infinitely long in the elongation direction and infinitely narrow in the normal direction. These characteristics remain consistent regardless of the image scale, which renders the aforementioned evaluation indices ineffective. Furthermore, given that the chosen target detection algorithm may exhibit performance limitations, as noted in [46], resulting in issues such as reduced detection accuracy and an elevated leakage detection rate, this study introduces a novel water level elevation solution. This solution is capable of achieving precise water level detection, even when the original target detection algorithm exhibits low detection accuracy, as referenced in [47]. The relative accuracy, Pa, of the predicted water level elevation and the actual water level elevation are used as the evaluation index, which is shown as Equation (12).

(12) $$p_{a}=\left(1-\frac{|h_{real}-H|}{H_{real}}\right)\times 100\%.$$

### 4.3. Implementation Details

In this study, we employed a blind super-segmentation method for super-resolution recovery of the dataset. This process was executed on an NVIDIA GeForce A30, (NVIDIA, Santa Clara, CA, USA), allowing the dataset to be processed in just 3–4 h. The training configuration for the enhanced Yolov5 was set to 200 epochs. Ultimately, we opted to conduct testing on a 12th Gen Intel® Core™ i5-12500H, (NVIDIA, Santa Clara, CA, USA) laptop. The overall model comprised 0.3 M parameters (a mere 1/30 of Yolov5’s approximately 7.5 M parameters). In terms of speed, image detection took just 0.1 ms (compared to Yolov5’s 8 ms, representing a fraction of 1/80). Specifically, the learning rate was set as 5 × 10^−5^ and a weight decay was 1 × 10^−4^. The epochs were set to 30 on the datasets. Throughout the entire training process, the model iterated 500 times to achieve optimal results.

### 4.4. Experimental Results

First, we used ${\bar{p}}_{a}$ as the evaluation index to complete the quantitative result evaluation, where ${\bar{p}}_{a}$ is the mean value of the above water level detection accuracy evaluation index; the results are shown in Table 1. “Q/S” represents the number of images for each scene. “SR” stands for the temporal super-resolution operation, while “SR + Imp. Yolov5” signifies the integration of super-resolution and the improved Yolov5. The mean values of the relative accuracy between the detected water level height and the real water level height were 92.9%, 96.3%, 96.7%, and 98.5%, respectively. The fusion of each method significantly contributed to enhancing water level detection and identification. Additionally, the algorithm model proposed in this study achieved a score in the evaluation indicators that, when compared to the original Yolov5, demonstrated an accuracy sufficient for practical engineering applications. This indicates that the method presented in this work is capable of effectively completing the task of water level detection in challenging scenes.

In addition, to validate the effectiveness of the proposed method in this work, we compared the proposed model with the existing models based on water level detection, and we compared it with the latest DetSegNet [33], PIDNet [35], ASFF [48], CAM-UNet [49], TRCAM- based methods under the same benchmarks of the computation and model training methods. UNet+SR [50] and DeepLabv3+SR [51] were compared. With the same operation of SR, using the improved Yolov5 model based on this work, the optimal results were, indeed, achieved in the four scenarios. The specific results are shown in Table 2.

### 4.5. Ablation Studies

**Figure 3:**
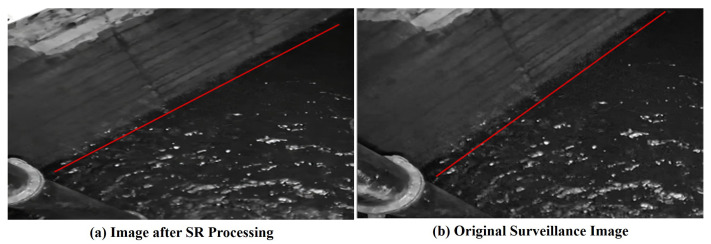
Comparison before and after super-resolution processing. The red line denotes the water level boundary. As illustrated in the figure, it becomes apparent that following the application of the super-resolution model, the water level line is rendered more clearly.

**Figure 4:**
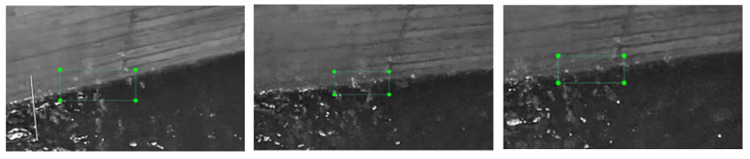
Water level boundary line. Upon being processed by super-resolution, the monitoring image exhibits a more defined boundary line on the water surface. The green box highlights the water level line that requires detection.

In addition, when the improved Yolov5 model proposed in this work is applied to water level detection, the water level line area must be marked with an anchor frame of a certain size, the specific effect of which is shown in Figure 3. The Yolov series model is mainly for target detection tasks, but as the water level line belongs to the steel target, using the traditional model based on target detection, it is difficult to obtain the essential characteristics of such a target effectively, so this work proposes a method based on the fusion of multi-scale features to effectively detect the critical state of the water level line to achieve a better characterization of target detection.Its detection visualization results are shown in Figure 4.

After the enhancement, Yolov5 has more advantages in water level line recognition: (1) the mesoscale feature mapping is added, which improves the ability of mesoscale feature mapping to recognize the water level line; (2) the earlier fusion of shallow feature mapping and deep feature mapping retains more spatial and detailed information, which makes the center of the prediction frame fit more closely to the water level line and increases the accuracy of the subsequently fitted straight line; (3) the splicing operation, convolution + residual operation, and convolution + batch normalization + activation function operation are reduced, which reduces the number of network layers and parameters, and is conducive to the lightness of the deep network.

**Figure 5:**
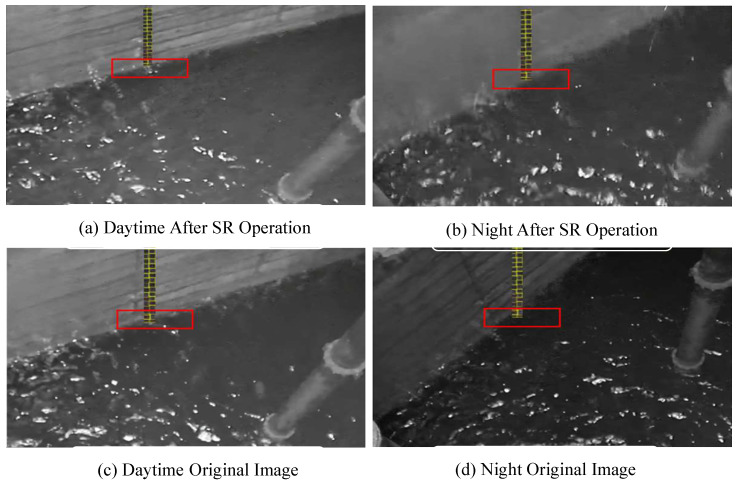
Improved monitoring result chart of Yolov5. The yellow elements depicted in the figure represent the binarized scale values. The red box highlights the recognition of its specific location.

Meanwhile, the detection results using the improved Yolov5 are presented in Figure 5. Specifically, Figure 5a,b depict the original monitoring images captured inside the catchment wells during daytime and nighttime, respectively. Figure 5c,d, on the other hand, show the images after undergoing the super-resolution (SR) operation. From these figures, it is evident that a clearer monitoring result can be achieved following the SR process.

**Figure 6:**
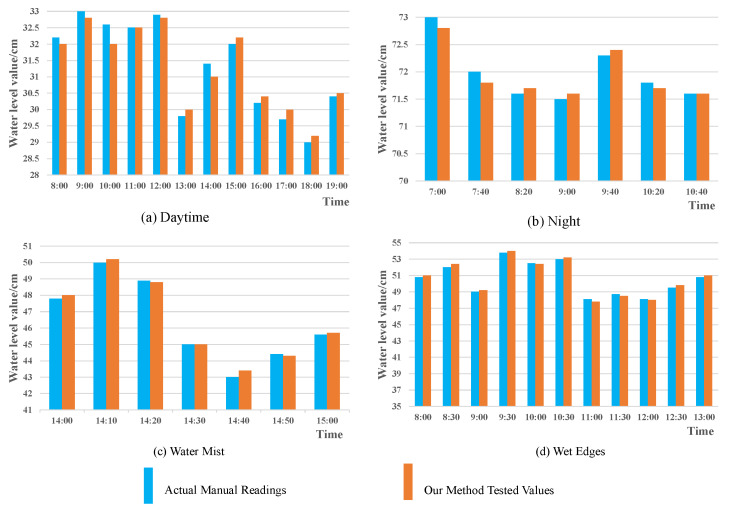
Comparison with manual readings.

To further validate its efficacy in engineering practice, we conducted artificial real-world measurements for comparison with the proposed method. The comparison results are presented in Figure 6. We compared the histograms by assessing the real-time water level in four scenarios. In these histograms, the red bars represent the water levels detected and recognized by the proposed method, while the blue bars depict the water levels manually read from the water level line. The maximum discrepancy between the detected water level and the manually read value is merely 0.8 cm. Both qualitative and quantitative experiments demonstrate the effectiveness of the proposed method in addressing the pertinent engineering practice problems.

**Figure 7:**
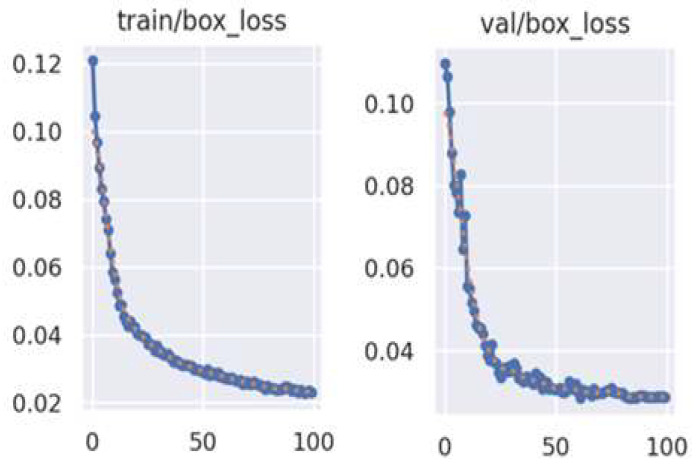
Curve change flowchart of loss functions.

To validate the training process of the method proposed in this work, we present the graphs depicting the loss function change curves of the model on both the training set and the validation set, as illustrated in Figure 7. Specifically, the graph on the left shows the loss change curve for the training set, while the graph on the right displays the corresponding change for the validation set. It is evident from the figure that the model proposed in this work generally adheres to the typical process of loss function variation.

Finally, we experimentally verify the specific value of the parameter λ, as shown in Table 3, from which it can be seen that the different values of the control parameter in the different scenarios have a relatively large impact on the experimental results. When the value of the parameter λ is 0.55, the optimal effect is obtained in all four scenarios. From this, we can analyze that when the control parameter is around 0.5, it can balance the effects of the two methods, achieve balanced optimization, and make the model reach the optimum.

### 4.6. Water Level Monitoring for Emerging Intelligent Systems Security Applications

**Figure 8:**
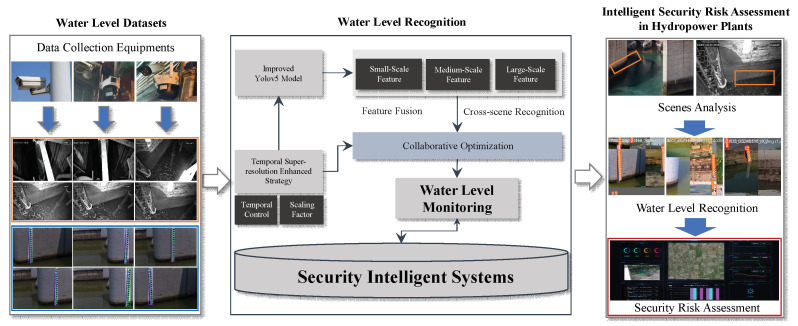
Applications of our algorithm to security intelligent systems.

Generally, intelligent water level monitoring is a widely used safety management method for modern hydropower plants. It can perform various safety risk assessment operations on the internal scenes of hydropower stations. Specifically, the integration of water level monitoring algorithms into new intelligent software and risk warning platforms is used to achieve rapid implementation of water level emergency warning systems. By using image super-resolution and the Yolov5 model in our method, our model has smaller parameters and faster execution efficiency compared to the latest models of the Yolo series (Yolov10, Yolov11). By using image recognition based on super-resolution enhancement processing, the recognition accuracy can be guaranteed. Therefore, the execution time complexity of our algorithm can reach the level of O(n), and it can recognize 4500 scene instances per second, which makes our algorithm a suitable choice for the application of intelligent system security risk assessment in hydropower plants. The specific deployment process is shown in Figure 8.

As illustrated in the figure above, the specific application process is primarily divided into three stages: water level dataset compilation, water level recognition, and intelligent security risk assessment. Initially, a range of surveillance devices are employed to gather water level data in indoor settings. For water level recognition, we utilize a temporal super-resolution strategy incorporating temporal control and a scaling factor approach to enhance the preprocessing of surveillance images. Subsequently, multi-scale (small, medium, and large) feature extraction based on the Yolov5 model is conducted on the images processed in the previous step. Following this, the two aforementioned modes are leveraged for collaborative training to accomplish water level monitoring. Upon completion of these processes, our algorithm is deployed within a security intelligent system, facilitating the analysis of scenes, water level recognition, and various other security risk assessments in hydropower plants.

In addition, by using image super-resolution and an improved Yolov5 model in our algorithm, it has fewer model parameters and faster algorithm execution speed compared to the latest Yolo series models (Yolov10, Yolov11). And the recognition of the image after super-resolution can maintain the accuracy. Thus, our algorithm has achieved good guarantees in both time complexity and accuracy, which allows it to be used well in specific intelligent security systems, including HT-for-Web (HTWEB, https://chinadaily.com.cn/, accessed on 19 December 2024) analysis software, and warning platforms for water level urgency in hydropower plants.

## 5. Conclusions, Limitations, and Future Work

### 5.1. Conclusions

This study introduced a water level monitoring framework based on a temporally enhanced image super-resolution technique and an improved Yolov5 model. Specifically, the former proposed a temporal super-resolution enhancement (scaling)-based technique to control different degrees of super-resolution images. The Yolov5 is improved by a multi-level feature fusion method with enhanced mesoscale features to capture the anchor frame of the water level line of a certain size, and the real water level line is approximated by fitting the center point of the anchor frame. Experimental results show that the proposed method can effectively detect the water level line inside the catchment well in real scenarios, and its detection accuracy provides reliable support for practical applications.

### 5.2. Limitations and Future Work

The dataset size proposed in this article has ample room for expansion. To broaden the diversity of the data distribution and enhance the model’s robustness for practical applications, it is necessary to incorporate more scenarios. Furthermore, the temporal super-resolution method’s challenge in precisely controlling the level of temporal enhancement often leads to inevitable recognition errors in practical applications. Consequently, addressing this challenge will be a key strategy in our future improvements.

## Figures and Tables

**Table 1:** Experimental results on 4 different scenes (${\bar{p}}_{a}$%).

Scenes	Q/S	w/o SR	Yolov5	w/SR	Imp. Yolov5	SR+Imp. Yolov5
Daytime	1800	**90.5**	**93.1**	**97.1**	**96.7**	**98.6**
Nighttime	2000	89.2	92.6	96.4	95.5	98.4
Mist	1000	88.5	92.1	95.9	97.7	97.7
Wet Edges	200	88.7	91.2	96.5	97.8	98.2
Average		89.2	92.2	96.5	97.0	98.2

**Table 2:** Experimental results for different models (${\bar{p}}_{a}$%).

Model	Scene			
	Daytime	Nighttime	Mist	Wet Edges
DetSegNet+SR [1]	96.2	95.1	94.2	95.2
PIDNet+SR [35]	92.1	92.3	93.2	93.1
ASFF+SR [48]	97.4	96.2	95.1	95.5
CAM-UNet+SR [49]	97.3	97.3	94.1	93.2
TRCAM-UNet+SR [50]	96.2	95.4	91.9	92.3
DeepLabv3+SR [51]	94.2	92.1	94.3	91.7
Ours	**98.6**	**98.4**	**97.7**	**98.2**

**Table 3:** Experimental results for different λ (${\bar{p}}_{a}$%).

λ	Scene			
	Daytime	Nighttime	Mist	Wet Edges
0.15	91.2	90.1	96.2	92.2
0.35	92.2	91.1	91.2	89.1
0.55	**98.6**	**98.4**	**97.7**	**98.2**
0.75	96.5	93.2	93.0	91.2
0.95	86.2	85.2	82.4	93.2

## Data Availability

The data presented in this study are available on request from the corresponding author.
